# Supplementary material for: Non-canonical interplay between glutamatergic NMDA and dopamine receptors shapes synaptogenesis
Source: Nat Commun. 2024 Jan 2;15:27. doi: 10.1038/s41467-023-44301-z (PMC10762086; doi:10.1038/s41467-023-44301-z)
Supplement: Supplementary file 1 — Supplementary information [file 41467_2023_44301_MOESM1_ESM.docx]

**Supplemental materials for**

**Non-canonical interplay between glutamatergic NMDA and dopamine receptors shapes synaptogenesis**

Nathan Bénac^1§^, G. Ezequiel Saraceno^1§^, Corey Butler^1§^, Nahoko Kuga^2,3^, Yuya Nishimura^2^, Taiki Yokoi^3^, Ping Su^4^, Takuya Sasaki^2,3^, Mar Petit-Pedrol^1^, Rémi Galland^1^, Vincent Studer^1^, Fang Liu^4^, Yuji Ikegaya^2,5,6^, Jean-Baptiste Sibarita^1^, Laurent Groc^1*^

# Affiliations

^1^ Univ. Bordeaux, CNRS, IINS, UMR 5297, F-33000 Bordeaux, France

^2^Laboratory of Chemical Pharmacology, Graduate School of Pharmaceutical Sciences, The University of Tokyo, 7-3-1 Hongo Bunkyo-ku, Tokyo, 113-0033, Japan

^3^Department of Pharmacology, Graduate School of Pharmaceutical Sciences, Tohoku University, 6-3 Aramaki-aoba, Sendai, Miyagi, 980-8578, Japan

^4^Campbell Family Mental Health Research Institute, Centre for Addiction and Mental Health, University of Toronto, Toronto, Canada

^5^Center for Information and Neural Networks, Suita City, Osaka, 565-0871, Japan

^6^Institute for AI and Beyond, The University of Tokyo, Tokyo 113-0033, Japan

^§^ These authors equally contributed

* Email: [laurent.groc@u-bordeaux.fr](mailto:laurent.groc@u-bordeaux.fr)

**
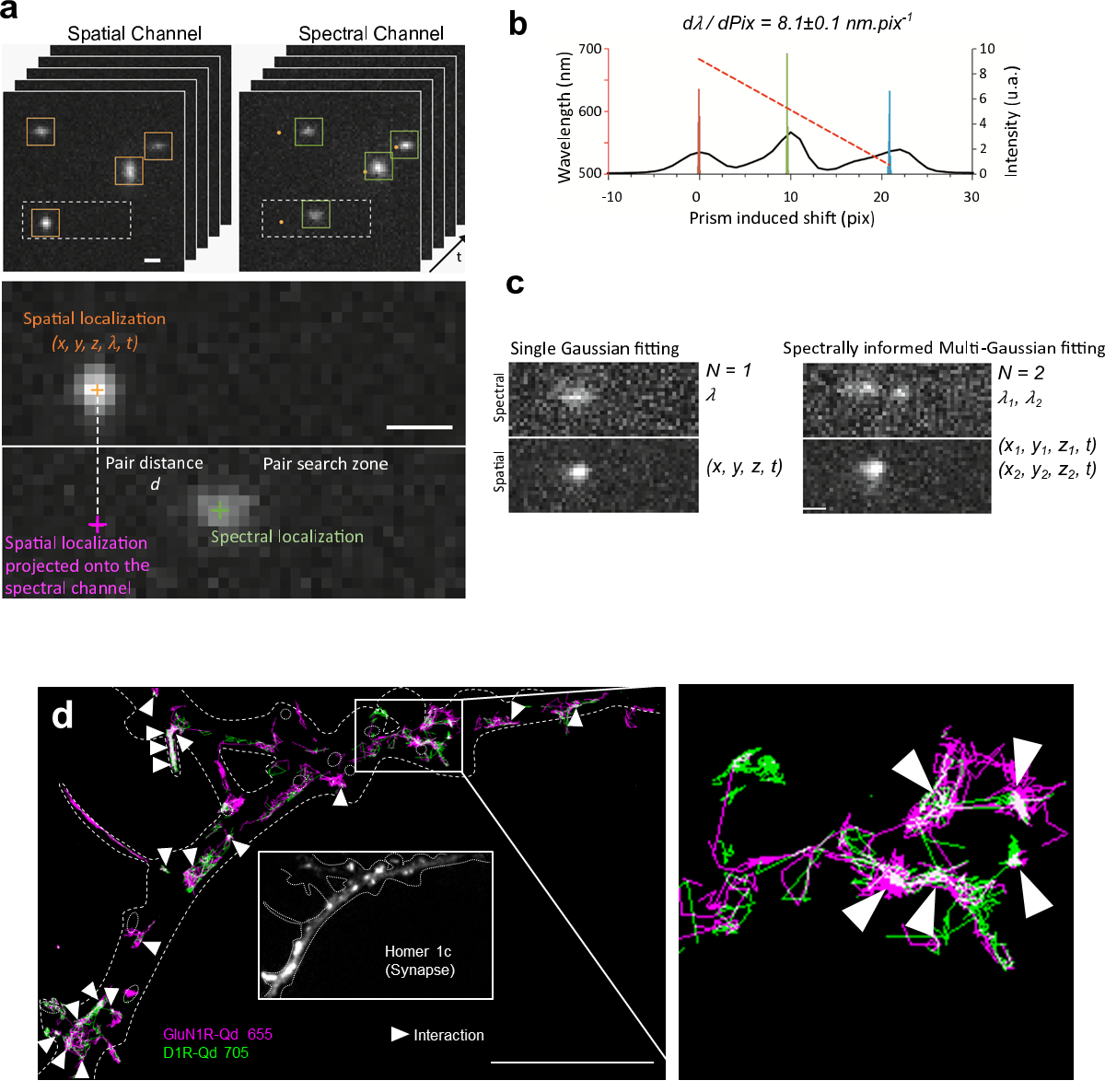
**

**Supplementary Fig. 1 MS-SMLM method. a** Spectrally displaced localization principle. **b** Calibration of the prism-induced shift in the spectral channel. **c**, Spectrally informed multi-gaussian fitting principle. **d** Representative reconstruction of GuN1-NMDAR and D1R surface diffusion. The white arrows represent the locations of the interacting events. Scale bar, 10 µm.


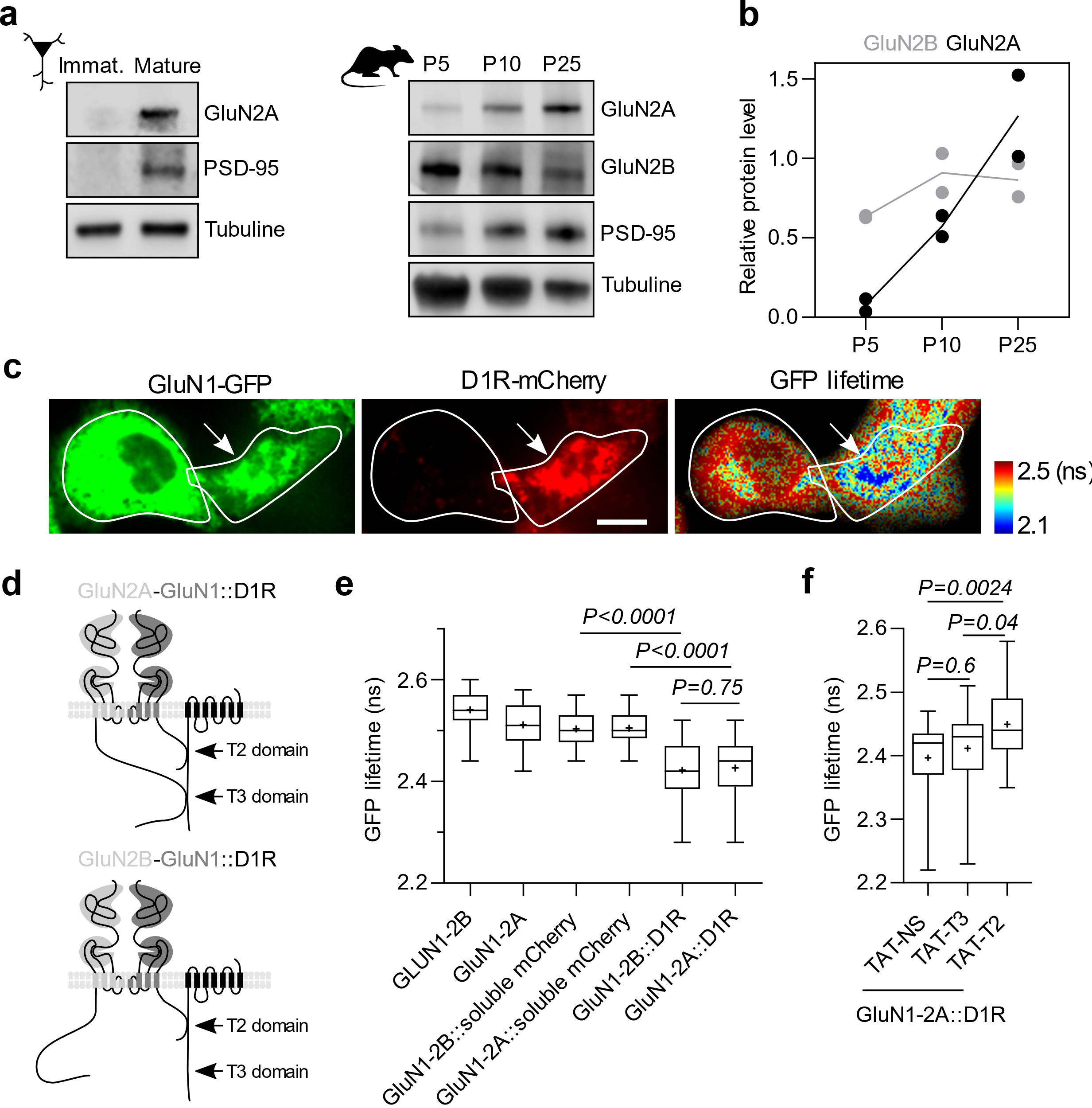
**Supplementary Fig. 2 Role of the GluN2A/B subunit in the GluN1-NMDAR-D1R interaction. a** Representative immunoblots with **b** corresponding quantification of the protein level (relative to tubuline) of the GluN2A and GluN2B subunits in the hippocampus of P5, P10 and P25 animals. N = 2 animals per group. **c** Representative fluorescence and color-coded GFP lifetime images. Scale bar, 50 µm. **d** Experimental set-up. **e** Quantification of the GluN1-GFP lifetime when GluN1-NMDAR is co-expressed, in COS-7 cells, either with the GluN2B (GluN1-2B, n = 31 cells) or GluN2A (GluN1-2A, n = 31 cells) subunits alone or together with soluble mCherry (acceptor control, GluN1-2B::soluble mCherry, n = 26 cells; GluN1-2A::soluble mCherry, n = 17 cells) or D1R-mCherry (GluN1-2B::D1R, n = 41 cells; GluN1-2A::D1R, n = 41 cells). Two-tailed unpaired t-test. **f** Quantification of the GluN1-GFP lifetime in the GluN1-GFP-GluN2A::D1RmCherry configuration after incubation with either a control competing TAT-peptide (TAT-NS, n = 29 cells), or targeting either the T2 domain (between GluN1-NMDAR and D1R; TAT-T2, n = 28 cells) or the T3 domain (between GluN2A-NMDAR and D1R; TAT-T3, n = 30 cells). One-way ANOVA with Tukey’s post-hoc test. Dots represent the mean.


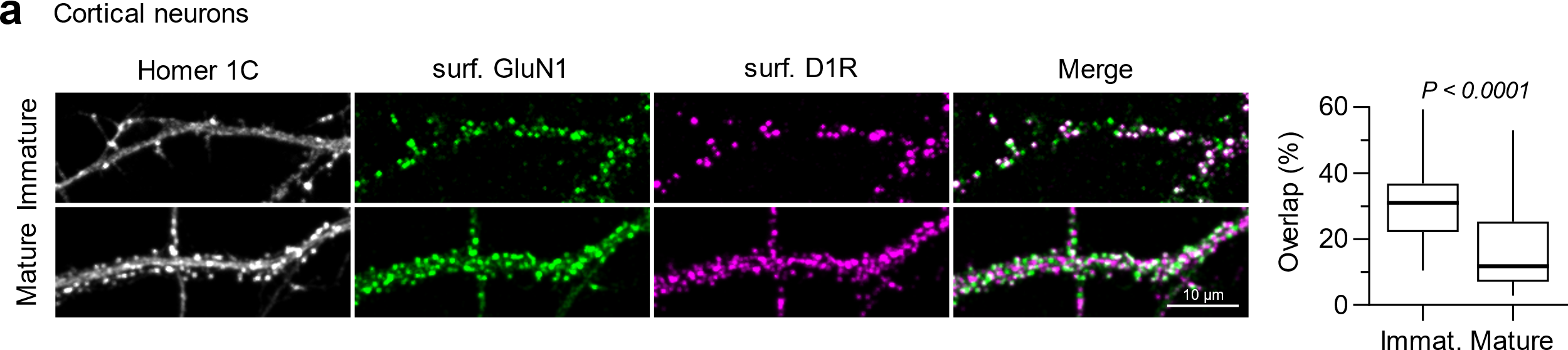
**Supplementary Fig. 3 GluN1-NMDAR-D1R interaction paradigm is conserved in cortical culture. a** Representative immunofluorescence images with quantification of GluN1-NMDAR-D1R overlap in immature (9 DIV, n = 25 cells) and mature (>15 DIV, n = 28 cells) cortical neurons. Two-tailed unpaired t-test. Scale bar, 10 µm.


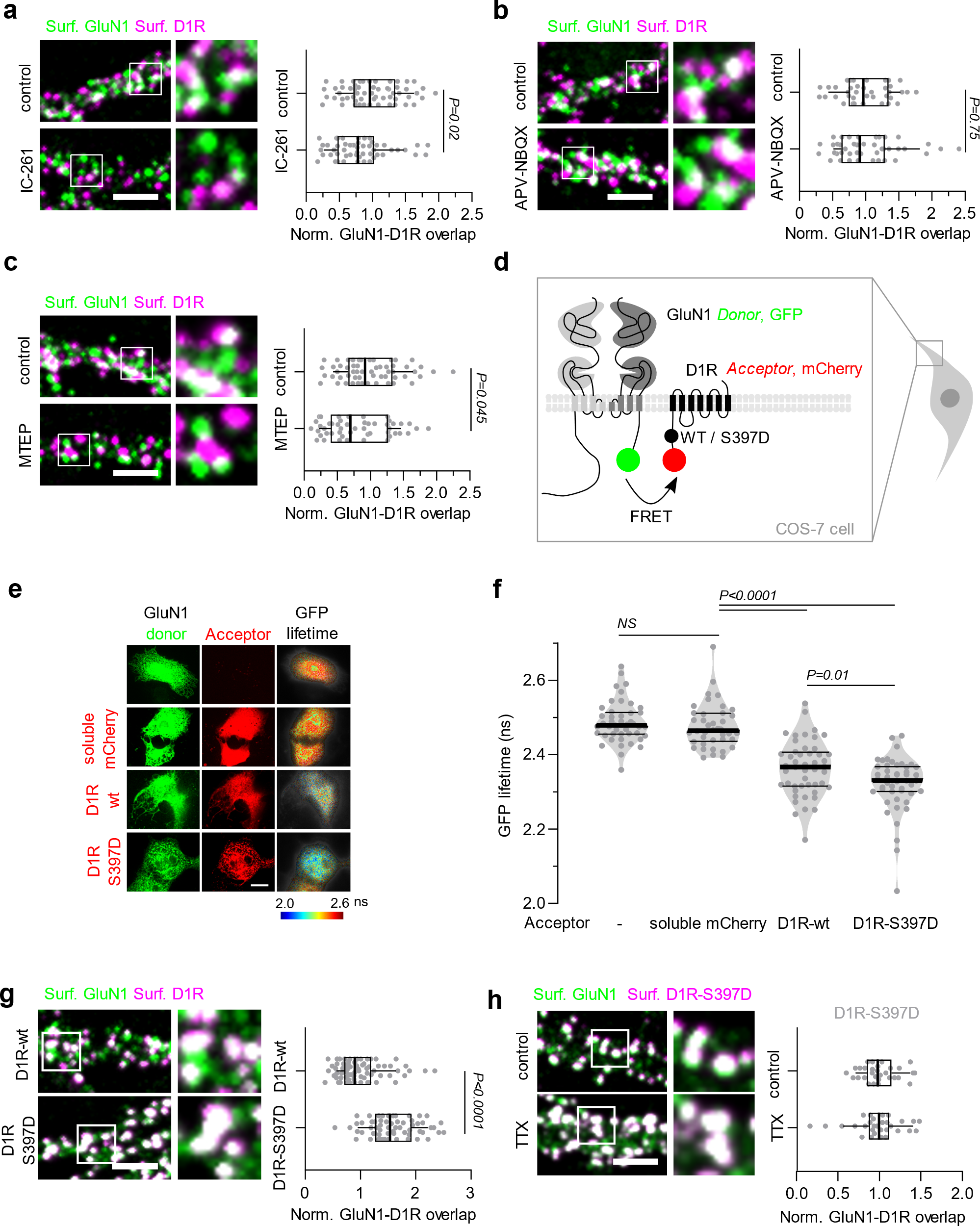
**Supplementary Fig. 4 GluN1-NMDAR-D1R is regulated in a CK1 and mGluR5-dependent manner. a-c** Representative images of hippocampal dendrites on which surface GluN1-NMDAR (green) and D1R (magenta) were labelled after exposure to (a) control (buffer; n = 59 fields) or IC-261 (CK1 inhibitor, 50 µM; n = 51 fields); (b) buffer (control; n = 38 fields) or AP-V and NBQX (50 µM and 2µM, respectively; n = 42 fields); (c) buffer (control, n = 56 fields) or MTEP (mGluR inhibitor, 10 µM; n = 49 fields). Two-tailed unpaired t-test. **d** Experimental set-up. **e** Representative fluorescence and color-coded GFP lifetime images. Scale bar, 20 µm. **f** Measure of GFP-fluorescence lifetime when GluN1-GFP is expressed alone (n = 46 cells) or together with soluble mCherry (control condition, n = 40 cells), D1R-wt-mCherry (n = 48 cells) or D1R-S397D-mCherry (47 cells), one-way ANOVA with Tukey’s post-hoc test. **g** Representative images of hippocampal dendrites on which surface GluN1-NMDAR (green) and D1R (WT or S397D, magenta) were labelled alongside quantification of the overlap between GluN1-NMDAR and D1R-wt (n = 25 cells, 52 fields) or D1R-S397D (n = 30 cells, 60 fields), Two-tailed unpaired t-test. Scale bar, 5 µm. **h** (left) Representative images of hippocampal dendrites on which surface GluN1-NMDAR (green) and D1R-S397D (magenta) were labelled after exposure to TTX; (right) quantifications of the overlap between GluN1-NMDAR and D1R-S397D following application of either buffer (CTL, n = 30 cells, 59 fields) or TTX (n = 30 cells, 53 fields). Two-tailed unpaired t-test. Scale bar, 5 µm.


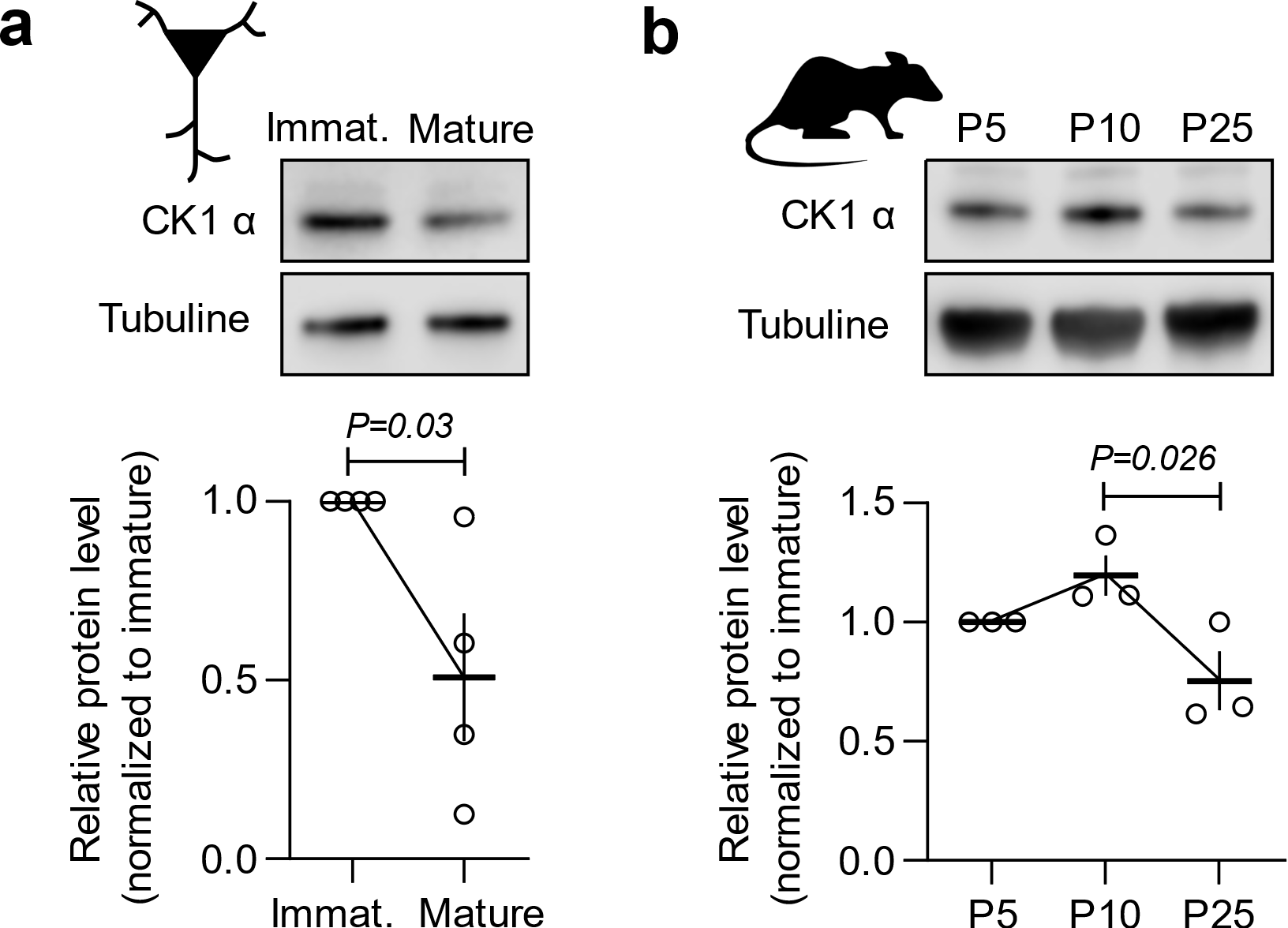


**Supplementary Fig. 5 CK1α is differentially expressed over neuronal development both *in vitro* and *in vivo*. a** Representative immunoblots with corresponding quantification of the relative protein level of CK1-alpha in immature (n=4) and mature (n=4) hippocampal cultures. The results are normalized to immature. Unpaired t-test. **b** Representative immunoblots with quantification of the relative protein level of CK1-alpha in hippocampi from P5, P10 and P25 animals. N = 3 animals per group. The results are normalized to P5. One-way ANOVA with Tukey’s post-hoc test.


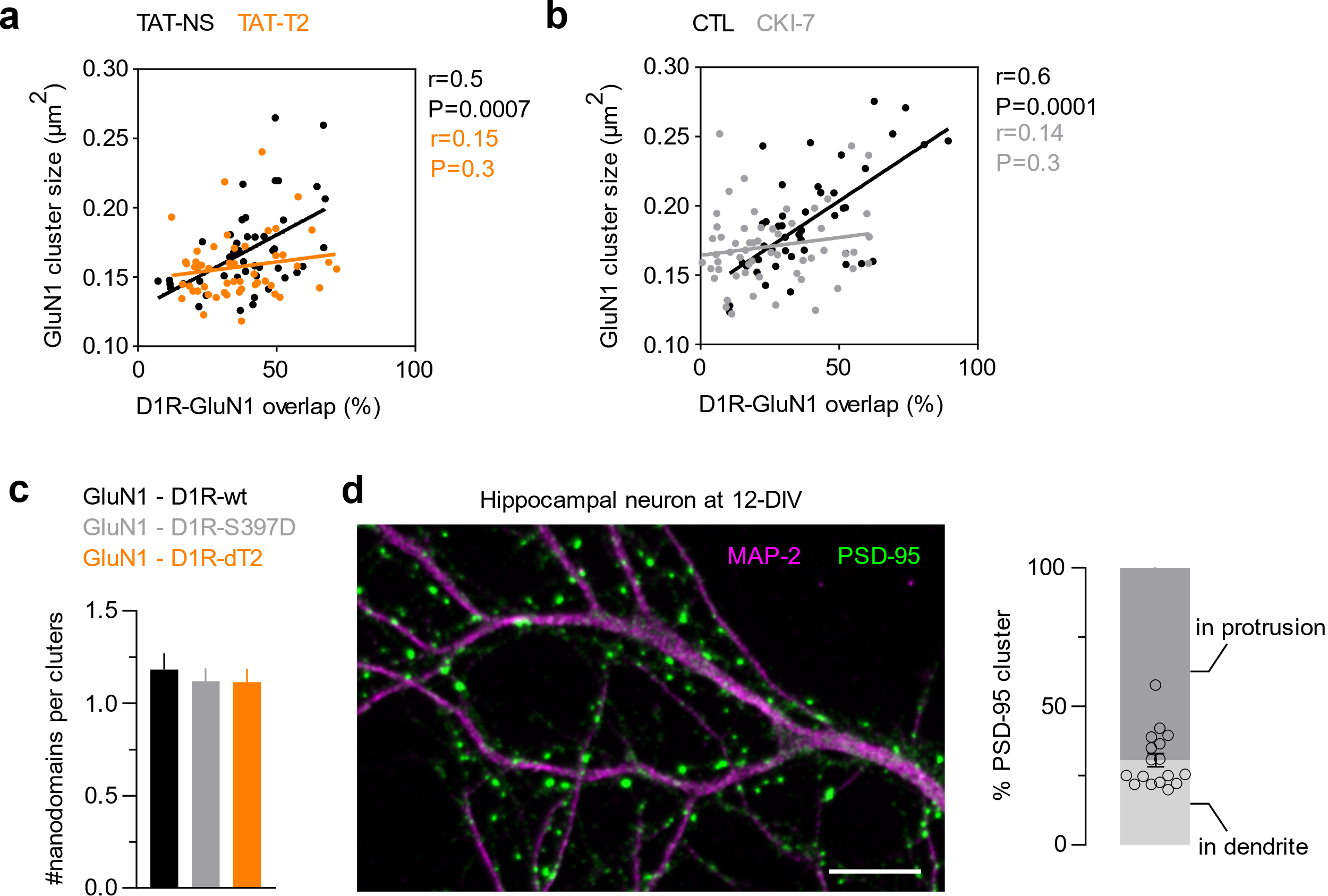
**Supplementary Fig. 6 The interaction between GluN1 and D1R controls the organization of GluN1 at the neuronal surface. a-b** Correlation between the size of GluN1-NMDAR cluster and the overlap between GluN1-NMDAR and D1R in control condition or after disruption of the interaction through competing peptides (a) or CKI-7 (b) incubation. **c** Quantification the number of GluN1 nanodomains per clusters when GluN1 is co-expressed with D1R-wt (n = 7 cells, 186 clusters and 214 nanodomains), D1R-dT2 (n = 7 cells, 217 clusters and 242 nanodomains) or D1R-S397D (n = 6 cells, 151 clusters and 169 nanodomains). **d** (left) Representative image of hippocampal dendrites at 12 days in vitro (DIV). Dendrites were labelled with MAP-2 (magenta) and postsynaptic densities with PSD-95 (green). Scale bar, 10 µm. (right) Percentage of post-synaptic densities i.e. PSD-95 puncta located in protrusion or onto the dendrite in DIV 12 hippocampal neurons (n = 17 neurons).


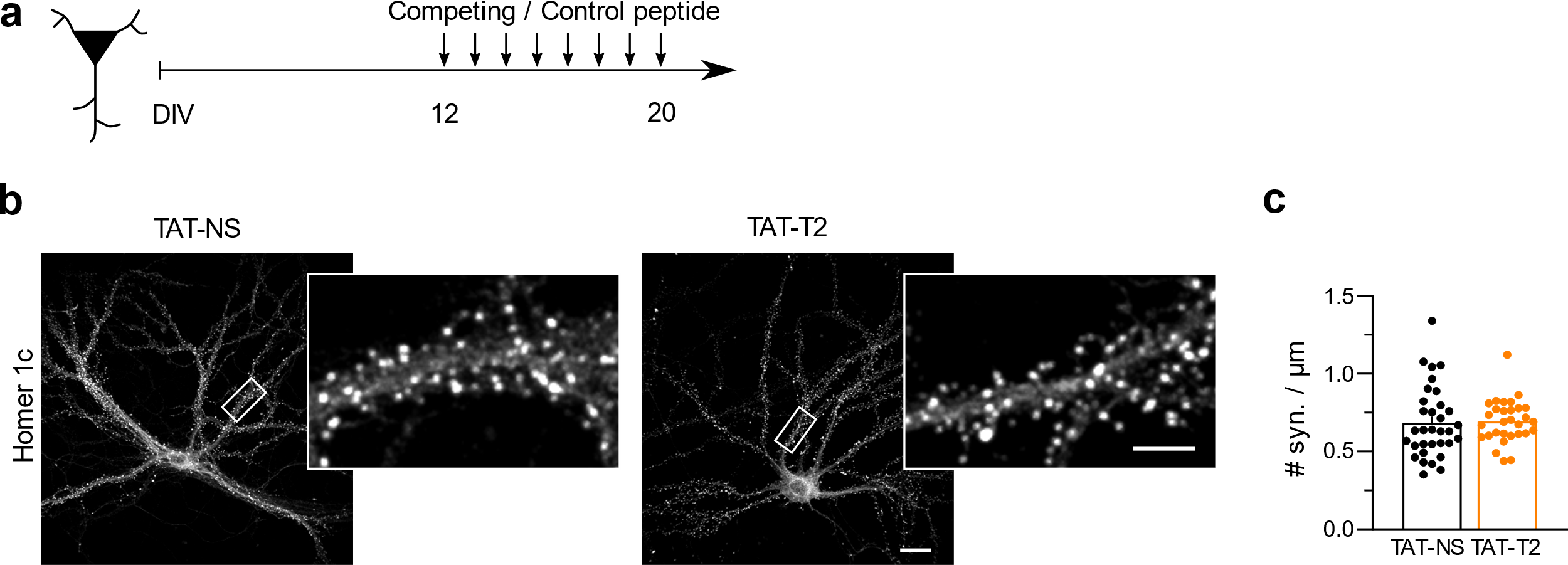
**Supplementary Fig. 7 Longer incubation of mature neurons with TAT-competing peptides does not alter the number of post-synapses in vitro. a** Experimental set-up. **b** Representative images of Homer 1c staining. Scale bar, 20 and 5 µm. **c** Quantification of the number of synapses in mature neurons treated for 8 consecutive days with control (TAT-NS, n = 33 cells) or competing peptides (TAT-T2, n = 30 cells). Two-sided unpaired t-test.


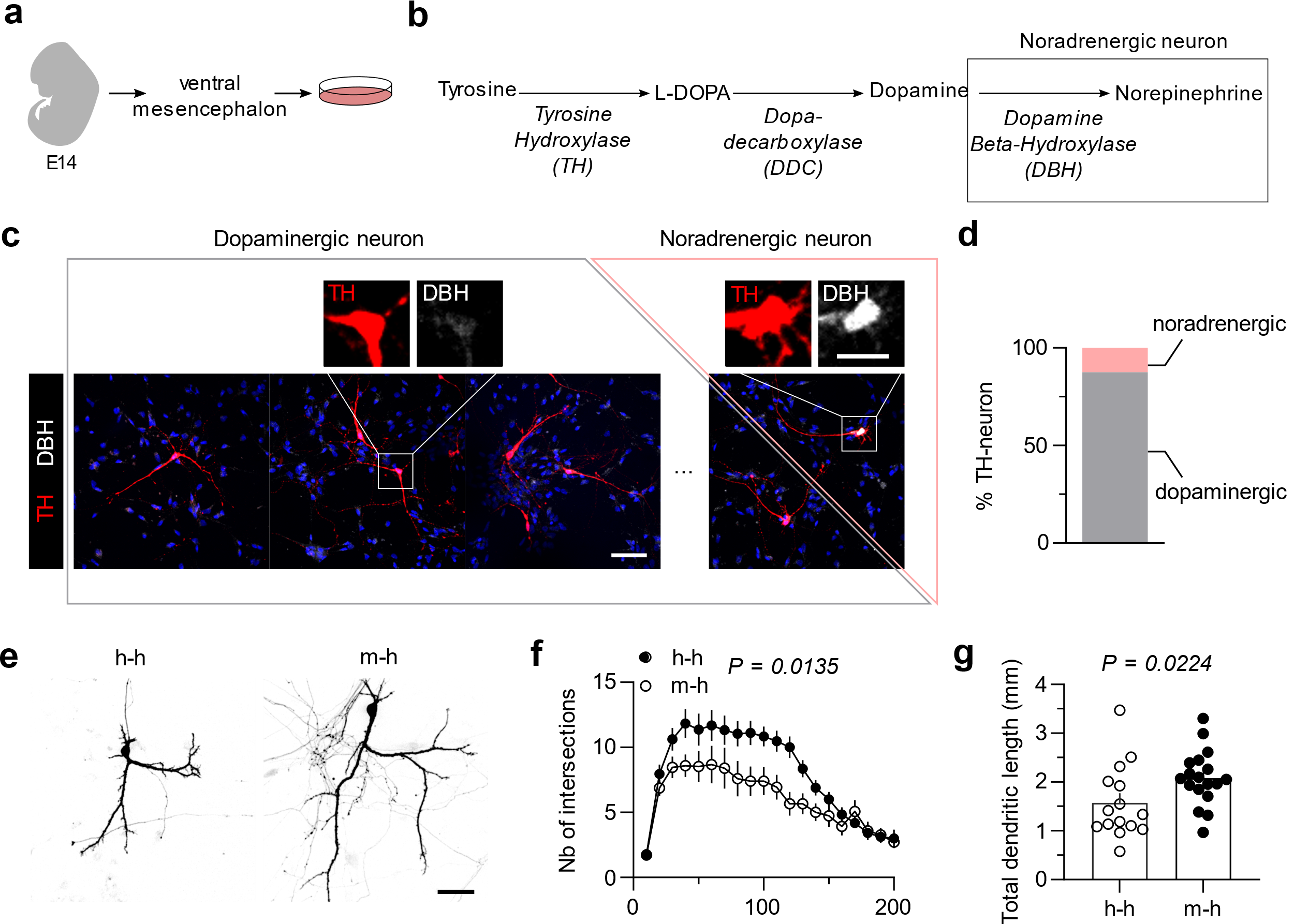
**Supplementary Fig. 8 Characterization of the primary neuronal culture from the midbrain. a** Experimental set-up. **b** Catecholamines synthesis pathway. **c** Representative fluorescence images of TH (red) and DBH (gray) staining. Scale bar, 50 and 20 µm. **d** Quantification of the percentage of dopaminergic (DBH negative, TH positive) and noradrenergic (DBH positive, TH positive) neurons. **e** Representative fluorescence images of hippocampal neurons co-cultured with hippocampal neurons (h-h) or dopaminergic-containing midbrain neurons (m-h). Scale bar, 50 µm; **f** Sholl Analysis (step size of 10 µm). Two-tailed Kolmogorov-Smirnov test. **g** Total dendritic size of hippocampal neurons co-cultured with hippocampal neurons (h-h n = 15 cells) or with midbrain neurons (m-h, n = 18 cells). Two-tailed unpaired t-test.
