# Supplementary material for: Non-canonical interplay between glutamatergic NMDA and dopamine receptors shapes synaptogenesis
Source: Nat Commun. 2024 Jan 2;15:27. doi: 10.1038/s41467-023-44301-z (PMC10762086; doi:10.1038/s41467-023-44301-z)
Supplement: Supplementary file 3 — Reporting Summary [file 41467_2023_44301_MOESM3_ESM.pdf]

nature portfolio

Corresponding author(s): Dr Laurent Groc

Last updated by author(s): Nov 27, 2023

Reporting Summary

Nature Portfolio wishes to improve the reproducibility of the work that we publish. This form provides structure for consistency and transparency in reporting. For further information on Nature Portfolio policies, see our Editorial Policies and the Editorial Policy Checklist.

Statistics

For all statistical analyses, confirm that the following items are present in the figure legend, table legend, main text, or Methods section.

n/a	Confirmed	
☐	☒	The exact sample size (*n*) for each experimental group/condition, given as a discrete number and unit of measurement
☐	☒	A statement on whether measurements were taken from distinct samples or whether the same sample was measured repeatedly
☐	☒	The statistical test(s) used AND whether they are one- or two-sided<br>*Only common tests should be described solely by name; describe more complex techniques in the Methods section.*
☐	☒	A description of all covariates tested
☐	☒	A description of any assumptions or corrections, such as tests of normality and adjustment for multiple comparisons
☐	☒	A full description of the statistical parameters including central tendency (e.g. means) or other basic estimates (e.g. regression coefficient) AND variation (e.g. standard deviation) or associated estimates of uncertainty (e.g. confidence intervals)
☐	☒	For null hypothesis testing, the test statistic (e.g. *F*, *t*, *r*) with confidence intervals, effect sizes, degrees of freedom and *P* value noted<br>*Give P values as exact values whenever suitable.*
☒	☐	For Bayesian analysis, information on the choice of priors and Markov chain Monte Carlo settings
☒	☐	For hierarchical and complex designs, identification of the appropriate level for tests and full reporting of outcomes
☒	☐	Estimates of effect sizes (e.g. Cohen's *d*, Pearson's *r*), indicating how they were calculated

Our web collection on statistics for biologists contains articles on many of the points above.

Software and code

Policy information about availability of computer code

Data collection	Metamorph was used to collect the microscopy data, Li-FLIM (v1.2.12) was used to collect the FRET data, image studio was used to collect the western blot data.
Data analysis	MS-SMLM movies were analyzed using PALMTracer (Butler et al., 2022), FRET images were analyzed with Li-FLIM (v1.2.12), Spinning disk confocal images were analyzed with Fiji software, dSTORM images were reconstructed with PalmTracer and analyzed with SR-Tesseler (Levet et al., 2015). Immunoblots were analyzed with Empiria software and imageJ (coIP), Electrophysiological and calcium data were analyzed with Matlab 2018a, All the graphs and statistical tests were performed using GraphPad Prism (v9.0)

For manuscripts utilizing custom algorithms or software that are central to the research but not yet described in published literature, software must be made available to editors and reviewers. We strongly encourage code deposition in a community repository (e.g. GitHub). See the Nature Portfolio guidelines for submitting code & software for further information.

Data

Policy information about availability of data

All manuscripts must include a data availability statement. This statement should provide the following information, where applicable:
- Accession codes, unique identifiers, or web links for publicly available datasets
- A description of any restrictions on data availability
- For clinical datasets or third party data, please ensure that the statement adheres to our policy

All raw and processed data will be made available upon request.

Human research participants

Policy information about studies involving human research participants and Sex and Gender in Research.

Reporting on sex and gender	N/A
Population characteristics	N/A
Recruitment	N/A
Ethics oversight	N/A

Note that full information on the approval of the study protocol must also be provided in the manuscript.

Field-specific reporting

Please select the one below that is the best fit for your research. If you are not sure, read the appropriate sections before making your selection.

☒ Life sciences ☐ Behavioural & social sciences ☐ Ecological, evolutionary & environmental sciences

For a reference copy of the document with all sections, see nature.com/documents/nr-reporting-summary-flat.pdf

Life sciences study design

All studies must disclose on these points even when the disclosure is negative.

Sample size	No statistical methods were used to predetermine sample size. Sample size was based on previous publications with similar models and experiments.
Data exclusions	No data were excluded from the analysis.
Replication	Every experiments have been successfully replicated between 3-4 times. The multidimensional spectral single molecule localization microscopy experiments have been performed by three independent experimentators.
Randomization	Cellular samples and animals were randomly allocated into experimental groups.
Blinding	Blinding was not suitable. However, to limit bias, quantifications were performed using a computational pipeline equally applied to all and the MS-SMLM experiments have been performed by three independent experimentators and analyzed using a computational pipeline.

Reporting for specific materials, systems and methods

We require information from authors about some types of materials, experimental systems and methods used in many studies. Here, indicate whether each material, system or method listed is relevant to your study. If you are not sure if a list item applies to your research, read the appropriate section before selecting a response.

Materials & experimental systems

n/a	Involved in the study	
☐	☒	Antibodies
☐	☒	Eukaryotic cell lines
☒	☐	Palaeontology and archaeology
☐	☒	Animals and other organisms
☒	☐	Clinical data
☒	☐	Dual use research of concern

Methods

n/a	Involved in the study	
☒	☐	ChIP-seq
☒	☐	Flow cytometry
☒	☐	MRI-based neuroimaging

Antibodies

Antibodies used

Primary antibodies:
rabbit polyclonal anti-GFP (A6455, ThermoFisher Sc., 1/500 or 1/10 000), mouse monoclonal anti-Flag (F1804, Sigma-Aldrich, 1/500 or 1/10 000), mouse monoclonal anti-TH (clone LNC1, MAB318, Merck Millipore, 1/1000), mouse anti-CK1 alpha (sc-74582, Santa Cruz, 1/1000), mouse monoclonal anti-beta tubulin (clone TUB2.1, C4585, Sigma Aldrich, 1/5000), rabbit anti-GluN2A (custom-made, Agrobio, 1/200), rabbit anti-GluN2B (custom-made, Agrobio, 1/200), rabbit anti-D1R (17934-1-AP, Proteintech, 2µg), rabbit anti-D2R (55084-1-AP, Proteintech, 2µg), rabbit anti-NR2A (NB300-105, Novus Biologicals, 1/1000), rabbit anti-NR2B (ab65783, Abcam, 1/1000), mouse anti-D2R (sc-5303, Santa Cruz Biotech, 1/500), rat monoclonal anti-D1R (D2944, Sigma-Aldrich, 1/1000), chicken anti-MAP2 (ab5392, Abcam, 1/5000), mouse anti-Synapsin-1 (106011, Synaptic System, 1/1000), mouse anti-PSD95 (MA1-046, ThermoFisher Sc., 1/500), rabbit anti-DBH (ab209487, Abcam, 1/2000).
Secondary Antibodies:
goat anti-mouse AF 488 (A11001, ThermoFisher Sc., 1/1000), Donkey anti-mouse AF 647 (A31571, ThermoFisher Sc., 1/1000), goat anti-rabbit AF 647 (A21244, ThermoFisher Sc., 1/1000), Goat anti-chicken AF 488 (A11039, ThermoFisher Sc., 1/1000), anti-mouse H +L HRP (715-035-150, Jackson Immunoresearch, 1/5000), anti-rabbit H+L HRP (711-035-152, Jackson Immunoresearch, 1/5000), goat-anti-rabbit IgG (H+L) highly cross-absorbed secondary antibody Alexa Fluor Plus 800 (A32735, ThermoFisher Sc., 1/5000), Alexa Fluor 790 AffiniPure Goat-anti-Rat IgG (light chain specific) (112-655-175, Jackson Immunoresearch, 1/1000), Alexa Fluor 790 AffiniPure Goat-anti-Rabbit IgG (light chain specific) (115-655-174, Jackson Immunoresearch, 1/1000).
Quantum Dots:
F (ab')2-Goat anti-Rabbit IgG-coupled Qdot655 (Q11422MP, ThermoFisher Sc., 1/50000), F (ab')2-Goat anti-Rabbit IgG-coupled Qdot705 (Q11461MP, ThermoFisher Sc., 1/50000).

Validation

Custom-made rabbit anti-GluN2B antibody was verified in Kellermayer et al., 2018 (PMID: 30269991) and has been used extensively in previous publications (Ferreira et al., 2020, Johansson et al., 2020).
Custom-made rabbit anti-GluN2A antibody was verified in Kellermayer et al., 2018 (PMID: 30269991) and has been used extensively in previous publications (Ferreira et al., 2020, Johansson et al., 2020).
Each antibody was validated for the resepctive species and application by the correspondent manufacturer.
GFP (https://www.thermofisher.com/antibody/product/GFP-Antibody-Polyclonal/A-6455)
Flag (https://www.sigmaaldrich.com/FR/fr/product/sigma/f1804)
TH (https://www.sigmaaldrich.com/FR/fr/product/mm/mab318)
CK1-alpha (https://www.scbt.com/fr/p/casein-kinase-ialpha-antibody-h-7?requestFrom=search)
CK1-delta (https://www.scbt.com/p/casein-kinase-idelta-antibody-c-8?requestFrom=search)
tubulin (https://www.sigmaaldrich.com/FR/fr/product/sigma/c4585)
D1R (https://www.ptglab.com/products/DRD1-Antibody-17934-1-AP.htm) https://www.sigmaaldrich.com/FR/fr/product/sigma/d2944)
D2R (https://www.ptglab.com/products/DRD2-Antibody-55084-1-AP.htm) https://www.scbt.com/p/d2dr-antibody-b-10?requestFrom=search)
NR2A (https://www.novusbio.com/products/nmdar2a-antibody_nb300-105)
MAP2 (https://www.abcam.com/map2-antibody-ab5392.html)
Synapsin-1 (https://sysy.com/product/106011)
PSD-95 (https://www.thermofisher.com/antibody/product/PSD-95-Antibody-clone-7E3-1B8-Monoclonal/MA1-046)

Eukaryotic cell lines

Policy information about cell lines and Sex and Gender in Research

Cell line source(s)

COS-7 cells were used for this study.

Authentication

COS-7 cell-line came directly from commercial sources that state for their authenticity (ECACC #87021302). We did not perform in-house identification.

Mycoplasma contamination

All cell lines were tested negative for mycoplasma contamination. Mycoplasma testing was performed by a third-party (Eurofins) via qPCR from cell culture media.

Commonly misidentified lines
(See ICLAC register)

No commonly misidentified cell lines were used.

Animals and other research organisms

Policy information about studies involving animals; ARRIVE guidelines recommended for reporting animal research, and Sex and Gender in Research

Laboratory animals	14 and 11 male C57BL/6j mice at P7 and P29 were used for the electrophysiological experiments. Sprague Dawley rats at P8 (n=3) and P36 (n=3) were used for the co-immunoprecipitation experiments. Sprague Dawley rats at P5 (n=3) were used for the IHC experiment. Pregnant Sprague Dawley rats at the age of 9-12 weeks old were purchased from Janvier Labs for the isolation of primary neurons. All animals were housed and maintained on a 12-h cycle at room temperature (22°C) and 40-70% (typically 60%) humidity with ad libitum access to food and water.
Wild animals	No wild animals were used in this study.
Reporting on sex	Tissue for dissociated hippocampal cultures was harvested from embryos of an unascertained mixture of sexes; males were used for in vivo and ex vivo experiments.
Field-collected samples	No field collected samples were used in this study.
Ethics oversight	This study was conducted in accordance with both the NIH and European Community guidelines (Directive 2010/63/EU) for the care and use of animals. The protocol was approved by the Experimental Animal Ethics Committee of the University of Tokyo (approval number: P29-14), by the Animal Care Committee of Centre for Addiction and Mental Health (approval number: #824) of the University of Toronto and by the local Bordeaux Ethics Committee (APAFIS#21727-2019010918359887).

Note that full information on the approval of the study protocol must also be provided in the manuscript.
